# Screening for atrial fibrillation and other arrhythmias in primary care

**DOI:** 10.1186/s12875-020-01151-8

**Published:** 2020-05-06

**Authors:** Kam Cheong Wong, Cindy Kok, Simone Marschner, Tim Usherwood, Clara K. Chow

**Affiliations:** 1 Westmead Applied Research Centre, Faculty of Medicine and Health, The University of Sydney, Westmead Hospital, Westmead, NSW Australia; 2 Westmead Clinical School, Faculty of Medicine and Health, The University of Sydney, Westmead, NSW Australia; 3 Bathurst Rural Clinical School, School of Medicine, Western Sydney University, Bathurst, NSW Australia; 4 School of Rural Health, Faculty of Medicine and Health, The University of Sydney, Orange, NSW Australia; 5 Northern Clinical School, Faculty of Medicine and Health, The University of Sydney, Royal North Shore Hospital, St Leonards, NSW Australia; 6 Department of Cardiology, Westmead Hospital, Westmead, NSW Australia

**Keywords:** Atrial fibrillation, Arrhythmia, Screening, Electrocardiography, Electrocardiogram, Mobile health, eHealth

## Abstract

**Background:**

Atrial fibrillation (AF) and other arrhythmias are prevalent and often encountered by general practitioners (GPs). In response to the growing prevalence and to assist practitioners in the diagnosis and management of AF, the Cardiac Society of Australia & New Zealand and Heart Foundation of Australia published the first Australian AF Guidelines in 2018. We aimed to examine (a) the proportion of GPs who performed any form of AF screening and identify the methods they applied, (b) GPs’ awareness of the AF Guidelines and approaches to arrhythmia screening, (c) the roles of conventional 12-lead ECG and mobile health devices, and (d) GPs’ confidence in ECG interpretation and need for training.

**Methods:**

A cross-sectional online survey titled “**GP**s **S**creen their patients for **A**trial **F**ibrillation and oth**E**r a**R**rhythmia (GPSAFER)” was conducted from October 2018 to March 2019. The participants were recruited via various GP networks across Australia. Ethics approval was granted by The University of Sydney.

**Results:**

A total of 463 surveys were completed. Many GPs (394/463, 85.1%, 95% CI 81.5–88.2%) performed some forms of AF screening and applied at least one AF screening method, most frequently pulse palpation (389/463, 84.0%). Some (299/463, 64.6%) GPs considered assessing their patients for other arrhythmias (237/299, 79.3% for complete heart block and 236/299, 78.9% for long-QT). Most GPs (424/463, 91.6%) were not using mobile ECG devices in their practice but some (147/463, 31.7%) were contemplating it. One third (175/463, 37.8%) of GPs were aware of the Australian AF Guidelines; those aware were more likely to perform AF screening (98.9% vs 76.7%, *p* <  0.001). Factors significantly and positively associated with AF screening were “awareness of the AF Guidelines” (*p* <  0.001), “number of years working in general practice” (*p* <  0.001), and “confidence in ECG interpretation of AF” (*p* = 0.003). Most GPs reported that they were very or extremely confident in interpreting AF (381/463, 82.3%) and complete heart block (266/463, 57.5%). Many GPs (349/463, 75.4%) would like to receive online ECG interpretation training.

**Conclusions:**

Assessment of arrhythmias is common in general practice and GPs are open to further training in ECG interpretation and using mobile ECG devices to aid their clinical practice. Increasing awareness of AF Guidelines and improving confidence in ECG interpretation may increase AF screening.

## Background

Atrial fibrillation (AF) is a common arrhythmia, with its prevalence estimated at 33.3 million people globally [1]. It is often encountered and managed by general practitioners (GPs) [2]. Some 80% of patients with suspected cardiac arrhythmias present to their GPs first [3]. AF can result in significant morbidity, accounting for a third of all strokes and may present symptomatically or asymptomatically [4]. Supraventricular tachycardia (SVT), conduction disorders, atrial and ventricular extrasystoles are also common, and can also present with palpitations, dizziness, syncope or asymptomatically [3, 5].

Early diagnosis of AF and treatment with anticoagulant medication can reduce the risk of stroke by over 60% [6]. However, 30% of those with persistent and permanent AF [7–9], and 40–70% of patients with paroxysmal AF are asymptomatic [7, 9, 10] and unaware of their condition and many are not receiving appropriate treatments. In response to the growing prevalence and gaps in treatment and to assist Australian practitioners in the diagnosis and management of AF, in 2018 the National Heart Foundation of Australia (NHFA) and Cardiac Society of Australia and New Zealand (CSANZ) published the first Australian AF Guidelines [11]: the Guidelines recommend that “Opportunistic annual screening for AF in general practice in patients aged 65 years or more is easily accomplished by pulse palpation, followed by an electrocardiogram (ECG) (if irregular), or by an ECG rhythm strip using a handheld ECG. This screening can be incorporated into standard consultations or undertaken by practice nurses during chronic care consultations or immunisations. Devices that provide a medical-quality ECG trace are preferred to pulse-taking or pulse-based devices (i.e. photoplethysmography and blood pressure oscillometry) for screening, because an ECG is required to confirm the diagnosis”.

These guidelines recommend GPs obtain a medical-quality electrocardiogram (ECG) trace and hence highlight the importance of GPs being able to interpret ECGs for AF and other common arrhythmias. Both conventional 12-lead ECG and single-lead handheld ECG are appropriate in this setting for screening AF [9].

We aimed to examine (a) the proportion of GPs who performed any form of AF screening and identify the methods they applied, (b) GPs’ awareness of the AF Guidelines and approaches to arrhythmia screening, (c) the roles of conventional 12-lead ECG and mobile health devices, and (d) GPs’ confidence in ECG interpretation and need for training.

## Methods

### Sampling and recruitment

This was a cross-sectional online survey aiming to recruit a diverse sample of GPs and GP registrars practising in urban and rural settings across Australia. The survey was distributed from 19th October 2018 to 31st March 2019 via GP networks and organisations. Participation was voluntary and required informed consent. We identified organisations with GP networks across Australia by searching the Australian Department of Health website [12] which provided links to Primary Health Networks (PHNs). We approached all 31 PHNs. In addition, we approached: the Australian College of Rural and Remote Medicine (ACRRM) and the Royal Australian College of General Practitioners (RACGP) and all their national, state, territory and rural entities (total nine entities), all nine Australian General Practice Training Organisations, 18 GP departments in Australian Universities that have school/ faculty of medicine, and the major GP peers groups including the General Practitioners Down Under (GPDU) (an authenticated GPs group in Facebook), the GP Supervisors Association, the GP Registrars Association and the Heart Foundation Australia. We requested each organisation to promote the survey at least once in their newsletter and/or website. When no response was received 3 weeks after the first contact, a reminder email was sent.

### Survey design and data collection

We designed a survey titled “**G**eneral **P**ractitioners’ **S**creen their patients for **A**trial **F**ibrillation and oth**E**r a**R**rhythmias (GPSAFER)” (see supplementary file). The following topics were investigated in the survey: arrhythmia screening, awareness of the Australian AF Guidelines, use of conventional 12-lead ECG and handheld single-lead ECG devices, and practitioners’ confidence in ECG interpretation and need for training. We asked how they usually screened asymptomatic patients for AF (i.e. select all applicable methods: clinical examination including pulse palpation, conventional 12-lead ECG, Holter monitor, mobile screening device, or never screen); how frequently they screened AF in asymptomatic patients with hypertension, diabetes, valvular heart disease, heart failure, stroke, and those prescribed antidepressants (never, two-yearly, yearly, six-monthly or more frequently); were they aware of the Australian AF Guidelines and had they incorporated the guidelines into their clinical practice for patients aged 65 years or older (all/ most/ some/ few/ no patients aged 65 years or older); how they usually obtained an ECG (i.e. select all applicable methods: used on-site 12-lead ECG machine, referred to pathology service/ cardiology service/ local hospital, used a mobile handheld ECG device, other); how time-consuming was obtaining a conventional 12-lead ECG (extremely/ very/ moderately/ slightly/ not time-consuming); did they use a mobile device to obtain a single-lead ECG on patients (regularly/ sometimes (such as six-monthly)/ occasionally/ never but contemplating using one/ never but not contemplating using one) and how confident were they (extremely/ very/ moderately/ mildly/ not confident) that a mobile handheld ECG device could provide adequate quality information to detect AF, complete heart block and prolonged QT; did they consider assessing their patients for arrhythmias other than AF and specified the type of arrhythmia/ conduction disorder (select all applicable: complete heart block, second degree heart block, left bundle branch block, trifascicular block, long-QT, other); how confident were they in diagnosing ECG abnormalities (extremely/ very/ moderately/ mildly/ not confident): AF, complete heart block, distinguished between Mobitz I and II second degree heart block, left bundle branch block, trifascicular block and prolonged QT-interval; were they aware of the automatic ECG interpretation provided by conventional 12-lead ECG machine and how helpful was it (extremely/ very/ moderately/ slightly/ not helpful); had they attended ECG interpretation training in the last 3 years, did they want to attend one and their preferred mode of training delivery (hardcopy such as RACGP Check magazine/ webinar (seminar via the internet)/ online education module/ face to face workshop/ other, specify). The initial survey questions were developed by the study team and piloted with six practitioners to assess suitability and clarity. Through consultations with GP peers the survey was substantially shortened to enable completion in 5 mins. and thereby minimise the GP's workload and maximise participation. Data collection also included participant's demographic information, the number of years working in general practice (less than one year/ 1–4 years/ 5–9 years/ 10–19 years/ 20 years and more), the number of hours per week working in general practice (1–8/ 9–16, 17–24/ 25–32/ 33–40/ > 40 h) and their practice location postcode. We converted postcodes to state/territory using JavaScript codes [13] and categorised the postcodes into urban and rural areas using the “Accessibility/ Remoteness Index of Australia (ARIA)” online converter provided by The University of Sydney [14]. The survey was administered through the Research Electronic Data Capture (REDCap) platform [15]. Ethics approval was granted by The University of Sydney (ref: 2018/740).

### Statistical analysis approach

Assuming a conservative estimation that 50% of GPs performed any form of AF screening, the study would require a sample size of 385 for estimating the expected proportion with 5% absolute precision and 95% confidence. That is, we would be 95% confident that between 45% and 55% of the GP population did some form of AF screening [16]. Categorical variables were analysed using Chi-square or Fisher Exact Tests. 95% confidence intervals (CI) for proportions were computed. Statistical significance level was set at *P*-value < 0.05. Analysis was performed using SPSS version 25 [17]. With reference to the literature on barriers and enablers for performing AF screening and to explore other potential factors that could predict practice of AF screening, we performed multivariate logistic regression by including the following factors: demographics of the participants (urban/ rural, GP experience (years in practice), working hours per week, GP roles (registrar/ fellows), gender), time pressure (“how time-consuming to obtain a conventional 12-lead ECG”), “awareness of the AF Guidelines”, “confidence in ECG interpretation of AF”, “confidence in the quality of information provided by handheld ECG device to detect AF”, “how helpful is the automatic 12-lead ECG interpretation”, “use of single-lead handheld ECG device”, and “use of conventional 12-lead ECG”, and “attended ECG training in the last three years”. Multivariate logistic regression was performed using SAS version 9.4 [18].

## Results

A total of 83 organisations were approached via emails. The response rate was 51.8% i.e. 43 of the organisations agreed to disseminate the survey via their electronic newsletters and websites; 32 (38.6%) didn’t reply to the first and second email requests, and 8 (9.6%) declined the request (Table 1).

**Table 1:** General practitioner-related organisations that distributed the survey

Organisations	Organisations that distributed surveys	Organisations that declined or did not respond	Total number of organisations/ entities
GP Colleges ^a^	7 (77.8%)	2 (22.2%)	9
Primary Health Networks	15 (48.4%)	16 (51.6%)	31
GP Registrars Training Organisations	4 (44.4%)	5 (55.6%)	9
GP Departments in Universities	8 (44.4%)	10 (55.6%)	18
GP Associations and Peer-groups ^b^	9 (56.2%)	7 (43.8%)	16
Total	43 (51.8%)	40 (48.2%)	83

^a^Included RACGP national, states, territory and rural entities, and ACRRM

^b^Included GPDU, GP Supervisors Association and GP Registrars Association

Regarding participants, 684 individuals accessed the survey-link and the completion rate was 68.1% i.e. 466 completed the survey. Three of the completed surveys were from non-GP participants and were excluded resulting in the analysis sample of 463. Of the 218 that accessed the link but didn’t complete the survey, 131/218 (60.1%) didn’t complete the consent question and 87/218 (39.9%) consented but didn’t complete the survey of whom 79 didn’t answer any questions at all and eight answered some questions but terminated before the demographic questions. These incomplete responses were excluded from analysis.

Among the 463 participants, 328/463 (70.8%) were female, 378/463 (81.6%) were fellows of RACGP or ACRRM, 265/463 (57.2%) had less than ten years work experience in general practice and 325/463 (70.2%) usually worked 17–40 h per week, and 209/463 (45.1%) worked in regional rural areas (Table 2). The median time taken to complete the 18-question survey was four minutes (interquartile range was three to six minutes). The majority (258/463, 55.7%) of participants first heard of the survey from the GPDU.

**Table 2:** Demographics of participants from urban and rural areas

Location of practice: ^a^	Urban (*n* = 254, 54.9%)	Rural areas ^b^ (*n* = 209, 45.1%)	Overall (*n* = 463)
Gender: Female	180 (70.9%)	148 (70.8%)	328 (70.8%)
Male	72 (28.3%)	59 (28.2%)	131 (28.3%)
Rather not say	2 (0.8%)	2 (1.0%)	4 (0.9%)
General Practice Qualifications:			
FRACGP	201 (79.1%)	130 (62.2%)	331 (71.5%)
FACRRM and FRACGP	2 (0.8%)	11 (5.3%)	13 (2.8%)
FACRRM	0 (0%)	8 (3.8%)	8 (1.7%)
GP Registrars	35 (13.8%)	50 (23.9%)	85 (18.4%)
Other	16 (6.3%)	10 (4.8%)	26 (5.6%%)
Number of years working in general practice:			
Less than 1 year	8 (3.1%)	17 (8.1%)	25 (5.4%)
1–4 years	70 (27.6%)	63 (30.1%)	133 (28.7%)
5–9 years	59 (23.2%)	48 (23.0%)	107 (23.1%)
10–19 years	54 (21.3%)	40 (19.1%)	94 (20.3%)
20 years and more	63 (24.8%)	41 (19.6%)	104 (22.5%)
Number of hours per week working in general practice			
1–16 h	37 (14.6%)	27 (12.9%)	64 (13.8%)
17–32 h	111 (43.7%)	72 (34.4%)	183 (39.5%)
33–40 h	66 (26.0%)	76 (36.4%)	142 (30.7%)
More than 40 h	40 (15.7%)	34 (16.3%)	74 (16.0%)

^a^New South Wales: 163, Queensland: 103, Victoria: 84, South Australia: 41, Western Australia: 36, Tasmania: 20, Northern Territory: 11, and Australian Capital Territory: 5

^b^Inner regional areas: 127 (27.4%), Outer regional areas: 58 (12.5%), Remote/ Very remote areas: 24 (5.2%)

### Arrhythmia screening

On questioning about the approach to screening asymptomatic patients with AF, 394 (85.1%, 95% CI 81.5–88.2%) reported that they performed some forms of AF screening and applied at least one AF screening method (i.e. clinical examination including pulse palpation, conventional 12-lead ECG, Holter monitor, mobile device or use of blood pressure device) and 69 (14.9%, 95% CI 11.8–18.5%) reported that they would not screen asymptomatic patients for AF. Among the 394 respondents, 151 (38.3%, 95% CI 33.5–43.3%) applied two or more AF screening methods. The AF screening methods reported were as follows: pulse-palpation (389, 84.0%, 95% CI 80.4–87.2%), 12-lead ECG (147, 31.7%, 95% CI 27.5–36.2%), Holter monitor (42, 9.1%, 95% CI 6.6–12.1%), mobile screening device (16, 3.5%, 95% CI 2.0–5.6%), and use of blood pressure measuring device to detect AF (5, 1.1%, 95% CI 0.4–2.5%).

**Fig. 1:**
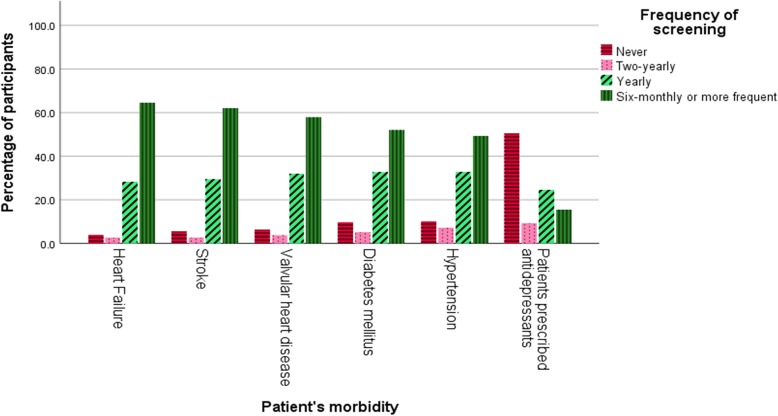
Frequency of atrial fibrillation screening by patients’ morbidities

GPs reported high rates of screening for AF for patients with comorbidities: patients with heart failure (95.9% screened), stroke (94.4% screened), valvular heart disease (93.7% screened), diabetes (90.1% screened), hypertension (89.8% screened) and patients prescribed antidepressant (49.5% screened) (Fig. 1).

Regarding other arrhythmias, 299/463 GPs (64.6%) reported they considered assessing their patients for arrhythmias other than AF. Among these, 237/299 (79.3%) assessed for complete heart block, 236/299 (78.9%) long-QT, 221/299 (73.9%) second degree heart block, 208/299 (69.6%) left bundle branch block, 99/299 (33.1%) trifascicular block, and 33/299 (11.0%) for other arrhythmias (supraventricular tachycardia, Wolff-Parkinson-White and ectopic beats etc).

### ECG applications and confidence in ECG interpretation

Respondents were asked to select all methods that they used to acquire an ECG: 416/463 (89.8%) obtained a 12-lead ECG in their practice, 110/463 (23.8%) referred to a pathology service, 14/463 (3.0%) referred to a cardiologist, 11/463 (2.4%) used a mobile handheld ECG device, 7/463 (1.5%) referred to hospital, and 2/463 (0.4%) used another method (e.g. ECG done by emergency department nurse). Many practitioners (214/463, 46.2%) found it was moderately to extremely time consuming to obtain a conventional 12-lead ECG. Most of them (456/463, 98.5%) were aware of the automatic ECG interpretation provided by a conventional 12-lead ECG machine but many of them (235/456, 51.5%) found the auto-interpretation slightly helpful or not helpful at all, while some of them (62/456, 13.6%) found it very or extremely helpful and remainder of them (159/456, 34.9%) found it moderately helpful.

When asked whether the practitioners used a mobile health device to obtain a single-lead ECG on patients, 424/463 (91.6%) never used one: 277/463 (59.8%) replied “never and not contemplating using one”, while 147/463 (31.7%) replied “never but contemplating using one”. However, some respondents reported that they were very or extremely confident that a mobile handheld ECG device could provide adequate quality of information to detect the following conditions: AF 104/463 (22.5%), complete heart block 52/463 (11.2%), and long-QT 30/463 (6.5%).

**Fig. 2:**
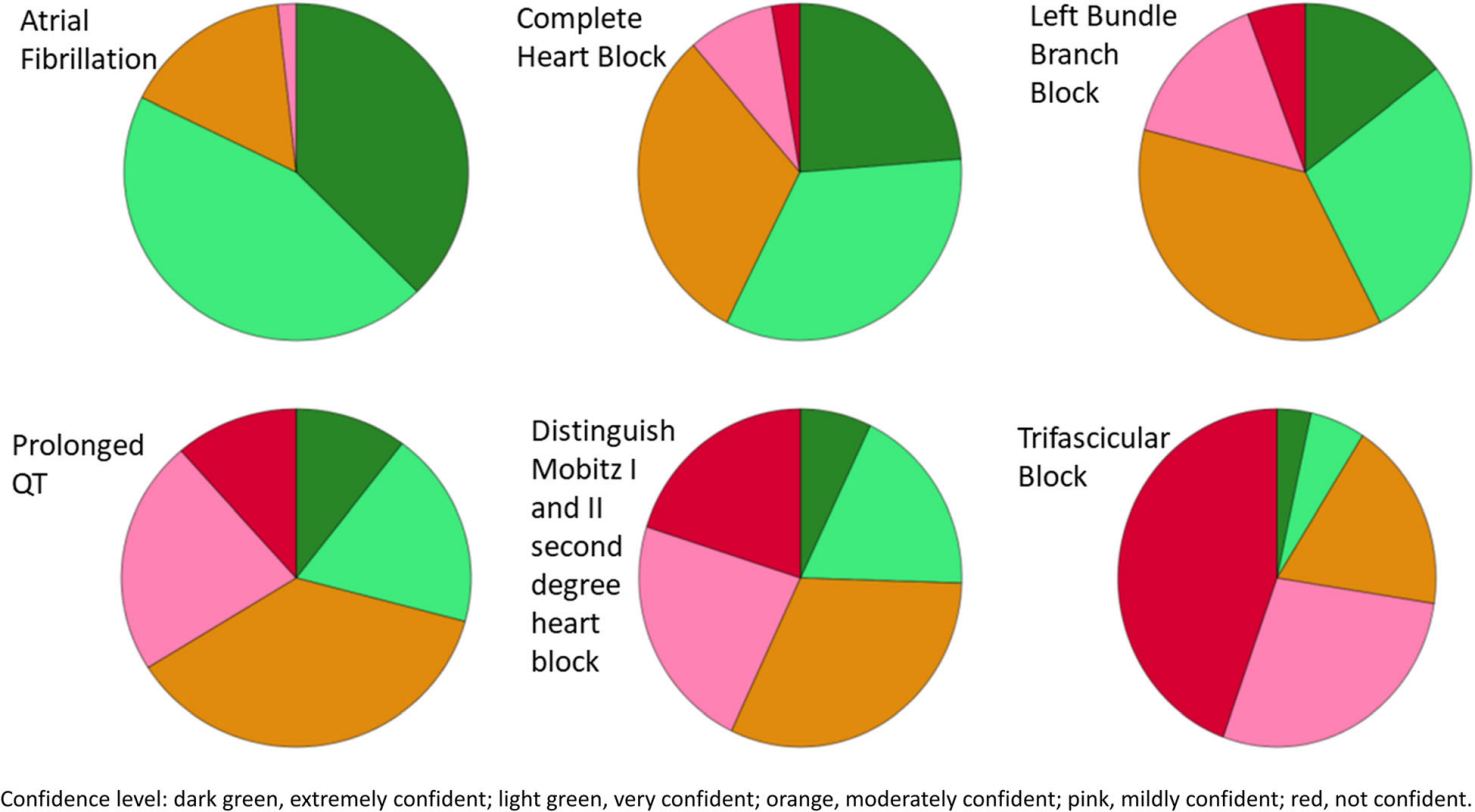
General practitioners’ confidence in interpreting electrocardiogram abnormalities

Only 110/463 practitioners (23.8%) reported that they had attended ECG interpretation training in the last 3 years. GPs reported that they were very or extremely confident in interpreting the following ECG abnormalities: AF (381/463, 82.3%), complete heart block (266/463, 57.5%), left bundle branch block (197/463, 42.5%), distinguishing between Mobitz I and II heart block (118/463, 25.5%), prolonged QT-interval (135/463, 29.2%) and trifascicular block (42/463, 9.1%) (Fig. 2). Many of them (349/463, 75.4%) would like to attend ECG interpretation training and their preferred mode of training were as follows: online training (239/463, 51.6%), face-to-face workshop (121/463, 26.1%), webinar (65/463, 14.0%) and RACGP Check magazine (31/463, 6.7%). About half of the participants (227/463, 49.0%) considered participating in an interview to discuss about mobile health devices or test use a mobile ECG device.

### Awareness of the Australian AF guidelines

The survey was launched approximately 2 months after the release of the Australian AF Guidelines and opened for participation for about 6 months. Approximately one third (175/463, 37.8%) of respondents were aware of the Australian AF Guidelines and among them 143/175 (81.7%) had applied the AF Guidelines with most or all their patients aged 65 years and older. Comparing those who were aware and unaware of the AF Guidelines, we found 98.9% versus 76.7% (*p* <  0.001) reported that they performed some forms of AF screening and applied at least one AF screening method and 46.9% versus 24.0% (*p* <  0.001) applied two or more AF screening methods (clinical examination including pulse palpation and device). Use of mobile screening devices was limited in both groups i.e. 12/175 (6.9%) and 4/288 (1.4%) (Table 3). Multivariate logistic regression analysis showed that factors significantly and positively associated with AF screening were “awareness of AF Guidelines” (*p* <  0.001), “number of years working in general practice” (*p* <  0.001), and “confidence in ECG interpretation of AF” (*p* = 0.003). Location of practice i.e. urban and rural (*p* = 0.360) and gender of GP (*p* = 0.510) were not significant predictors.

**Table 3:** Atrial fibrillation screening among those aware and not aware of Australian Atrial Fibrillation Guidelines

Screening practice and methods	Aware of guidelines (*n* = 175, 37.8%)	Not aware of guidelines (*n* = 288, 62.2%)	P-value
Did not perform asymptomatic AF screening	2 (1.1%)	67 (23.3%)	< 0.001
Performed AF screening with at least one AF screening method	173 (98.9%)	221 (76.7%)	< 0.001
Performed AF screening with two or more methods	82 (46.9%)	69 (24.0%)	< 0.001
Screening for AF with pulse palpation	171 (97.7%)	218 (75.7%)	< 0.001
Screening for AF with 12-lead ECG	76 (43.4%)	71 (24.7%)	< 0.001
Screening for AF with mobile device	12 (6.9%)	4 (1.4%)	0.003*

*Fisher exact test

## Discussion

We identified that most GPs performed some forms of AF screening and applied at least one method to screen for AF in clinical practice. Reported frequency of screening for AF was highest (> 90%) in patients with heart failure, stroke and valvular disease. Screening for AF was significantly and positively influenced by awareness of the AF Guidelines, the number of years working in general practice and their confidence in ECG interpretation of AF. Taggar and colleagues reported that improving practitioners’ skill in diagnosing AF had a role in AF screening [19]. Our finding of positive association between “confidence in ECG interpretation of AF” and “practice of AF screening” is consistent with their finding.

In 2017, the Economist Intelligent Unit (EIU) [20] examined how opportunistic screening for AF was applied in 20 countries by conducting an online survey on 1000 physicians (50 physicians in each country including Australia). Their survey aimed to identify the proportion of patients screened for AF in a primary care setting. They did not examine the association between the proportion of patients screened with the awareness of the Australian AF Guidelines. They reported that the proportion of patients aged ≥65 years screened for AF varied from 5 to 42% (11% in Australia). In our survey, we investigated the proportion of GPs performed any form of AF screening and identified the methods they used. We found that 394/463 (85.1%) of the practitioners performed some forms of AF screening and applied at least one AF screening method i.e. mostly pulse palpation (389/463, 84.0%). One third of the respondents (175/463, 37.8%) were aware of the Australian AF Guidelines and among them 143/175 (81.7%) had applied the AF Guidelines with most or all their patients aged 65 years and older. Though our results were not directly comparable with the EIU study, they were complementary i.e. while we didn’t determine how common AF screening was for people aged ≥65 years generally, we did determine what AF screening methods were utilised.

Despite mobile devices being referred to in the AF Guidelines, only 3.5% used a mobile device in practice, though about a third were contemplating using one, and about 23% were confident that a mobile device could provide adequate quality of information to detect AF. Obtaining an ECG trace is important in confirming the diagnosis of AF and about 90% of GPs reported being able to obtain a 12-lead ECG in their practice. However, GPs reported that they infrequently used 12-lead ECG for screening. Nearly half of GPs reported that 12-lead ECGs were time consuming, only about 80% reported they were confident in interpreting AF on ECGs and about half reported that ECG auto-interpretation was unhelpful or minimally helpful. These observations reflect some barriers to GPs using ECG to aid their AF screening.

Mobile health devices are emerging technologies. Yet, the rising availability and interest from consumers in mobile ECG devices [9, 21] coupled with references to these devices in current AF Guidelines [11] and screening for AF appears to fulfil the essential criteria for validity of screening programs [22], is in stark contrast to the very few GP respondents (3.5%) who reported using a mobile ECG device in AF screening. Nonetheless, about a third of GPs were contemplating the use of these devices. About a half of the respondents reported that automatic ECG interpretation on conventional 12-lead ECG machines were minimally helpful, which could be related to their concerns about the accuracy of the automatic ECG interpretation provided by the conventional 12-lead ECG [23, 24]. The lack of adoption of these technologies could be due to inertia to change practices which involves multi-factorial interactive processes such as practitioners’ knowledge of the new technologies, the perceived compatibility of the technologies with the current ones, the perceived usefulness, ease of use of the new technologies [25], time pressure, competing priorities, and technological problems [26, 27]. It may take time to instil greater awareness and improve confidence among practitioners to using newer technologies. Further research could help better understand the barriers and enablers to obtaining a medical-quality ECG trace and the role of mobile health devices to aid this in primary care [9, 26, 27].

With the increasing pressure on general practice in Australia, and indeed on primary care internationally, it is not surprising that time is a barrier to obtaining an ECG trace. In a study conducted by Somerville and colleagues, the average time taken to obtain a conventional 12-lead ECG in general practice was 10.6 min (including the time taken for preparing the patient and placing electrodes correctly) [28]. While time pressure could be a barrier to using conventional 12-lead ECG as a screening tool in a busy general practice setting, our survey findings indicated that when an ECG was required for a patient (including for diagnostic purpose), most GPs had access to conventional 12-lead ECGs at their practice. This observation was similar to a cross-sectional survey in a general practice setting conducted in Nottingham, UK [19]. The availability of conventional 12-lead ECG in general practice settings provides an opportunity for educators and researchers to design interventions to improve the efficiency and workflow of using the device in clinical practice.

Patients with other arrhythmias could present with similar symptoms to AF e.g. palpitations, syncope and dizziness. Hence, it is important that GPs are capable of diagnosing AF and other arrhythmias. Our findings show that most GP respondents were confident in diagnosing AF using ECGs. While several GPs considered assessing their patients for other arrhythmias, there was lower confidence in interpreting these less common but critical ECG abnormalities such as heart blocks and prolonged QT. Some guidelines do recommend ECG screening for prolonged QT in patients prescribed tricyclic antidepressants [29]. At present, these abnormalities are mainly diagnosed using conventional 12-lead ECG. There is lack of evidence of diagnostic accuracy of mobile health devices in diagnosing these other arrhythmias. Practitioners could apply conventional 12-lead ECG to diagnose these arrhythmias while more research to improve the diagnostic accuracy of mobile health device is underway. Few participants had attended recent ECG interpretation training; however, many were keen to attend training and most of them preferred ECG online training. This information supports ECG interpretation training being a needed feature of continuing medical education programs.

### Limitations

Recruiting GP participants in research has been reported by several studies as challenging [30, 31]. Our sampling methods could not obtain a strictly representative sample of all GPs across Australia. However, we managed to recruit participants from a wide geographical distribution covering urban and rural regional areas across Australia. The sample of 463 was larger than the minimum sample size of 385 required to investigate our first objective i.e. to examine the proportion of GPs that performed any form of AF screening. We acknowledge the potential of non-responder bias and that the survey reported GPs’ perceptions which might not reflect their true practice. However, our findings could lead to further research to examine the barriers and enablers for AF screening.

We limited the number of questions and cut questions during our pilot as we wanted the survey to take less than five minutes to complete. Therefore our study did not obtain information on the accuracy of ECG interpretation, the correlation between confidence in and correctness of ECG interpretation and the further details about the types of patients they actually saw in practice, and the actual frequency of AF screening among patients seen in their practice.

## Conclusions

Many GPs performed some forms of AF screening and applied at least one AF screening method, but there were barriers to the timely and efficient acquisition of medical-quality ECG traces to aid their diagnoses and a lack of uptake of newer technologies that could help to address these time barriers. Our findings suggest that raising awareness of AF Guidelines and improving confidence in ECG interpretation may increase uptake of AF screening. Also identifying and researching ways to enable efficient AF screening in primary care may assist. GPs were interested in ECG interpretation and keen for further knowledge, especially in the interpretation of other ECG abnormalities, and hence supporting this with accessible continuing education programs should be prioritised.

## Supplementary information

**Additional file 1.** General Practitioners Screen their patients for Atrial Fibrillation and othEr aRrhythmias (GPSAFER) – Survey questionnaire.

## Data Availability

The survey questionnaire was included as supplementary material. However, the survey dataset was not publicly available because ethics approval did not include providing the dataset publicly available.

## Abbreviations

ACRRM: Australian College of Rural and Remote Medicine
AF: atrial fibrillation
CI: confidence interval
CSANZ: Cardiac Society of Australia and New Zealand
ECG: electrocardiogram
GP: general practitioner
GPDU: General Practitioners Down Under
GPSAFER: **GP**s **S**creen their patients for **A**trial **F**ibrillation and oth**E**r a**R**rhythmia
NHFA: National Heart Foundation of Australia
PHN: Primary Health Networks
RACGP: Royal Australian College of General Practitioners
REDCap: Research Electronic Data Capture
SVT: Supraventricular tachycardia
