# Supplementary material for: Screening for atrial fibrillation and other arrhythmias in primary care
Source: BMC Fam Pract. 2020 May 6;21:79. doi: 10.1186/s12875-020-01151-8 (PMC7201749; doi:10.1186/s12875-020-01151-8)
Supplement: Supplementary file 1 — Additional file 1. General Practitioners Screen their patients for Atrial Fibrillation and othEr aRrhythmias (GPSAFER) – Survey questionnaire. [file 12875_2020_1151_MOESM1_ESM.docx]

**General Practitioners Screen their patients for Atrial Fibrillation and othEr aRrhythmias (GPSAFER)**



**Introduction**

Atrial fibrillation (AF) and other arrhythmias are common medical problems of increasing prevalence which are often identified and managed by GPs. The aim of this survey is to explore the views, knowledge and practices of general practitioners regarding cardiac arrhythmias, and additionally to explore the role of mobile devices in screening. This survey will take approximately 5 minutes to complete. As a token of appreciation, three participants who complete this survey will each receive a $50 gift card via a lucky draw.



This research will contribute to PhD studies of Dr Kam Wong (GP and PhD candidate, University of Sydney; Senior Lecturer in General Practice, Western Sydney University). His supervisors are Professor Clara Chow (Cardiologist and Professor of Medicine, University of Sydney), Professor Tim Usherwood (Professor of General Practice, University of Sydney) and Dr Cindy Kok (Honorary Researcher, University of Sydney).

| Please read the Participant Information Statement which provides detailed information about the study and your participation.  Do you consent to participate in this survey? Yes/ No  (When the participant answers ‘yes’ to the above question, he/ she will be taken to the survey questionnaire. If the participant answers ‘no’, he/she will exit the survey.) |
| --- |


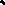


1. How do you usually screen asymptomatic patients for atrial fibrillation? [Please select all that apply. If you select “I never screen asymptomatic patient”, please leave all other options blank.]

[ ] I never screen asymptomatic patient

[ ] Clinical examination including pulse palpation

[ ] Conventional 12-lead ECG machine

[ ] Holter monitor

[ ] A mobile screening device

[ ] Other, please specify __________________________

(Participants who answer “I never screen asymptomatic patients” will skip the following Q2)

1. How frequently do you screen for atrial fibrillation in asymptomatic patients in the following patient groups? *[Note: This question is inquiring about the typical frequency with which you screen, not whether you do so systematically or opportunistically. The method of screening is whatever method you usually use.]*
2. Patients with hypertension

| Never | Two-yearly | Yearly | Six-monthly or more frequently |
| --- | --- | --- | --- |

1. Patients with diabetes

| Never | Two-yearly | Yearly | Six-monthly or more frequently |
| --- | --- | --- | --- |

1. Patients with a history of valvular heart disease

| Never | Two-yearly | Yearly | Six-monthly or more frequently |
| --- | --- | --- | --- |

1. Patients with a history of heart failure

| Never | Two-yearly | Yearly | Six-monthly or more frequently |
| --- | --- | --- | --- |

1. Patients with a history of stroke

| Never | Two-yearly | Yearly | Six-monthly or more frequently |
| --- | --- | --- | --- |

1. Patients prescribed antidepressant

| Never | Two-yearly | Yearly | Six-monthly or more frequently |
| --- | --- | --- | --- |

1. Are you aware of the Australian guidelines (2018 AF-guidelines developed by the National Heart Foundation of Australia in partnership with the Cardiac Society of Australia and New Zealand) recommending opportunistic screening for atrial fibrillation in people aged 65 years or older? Yes/ No

If yes, please select one of the following:

I have incorporated the guidelines into my clinical practice for:

[ ] all patients aged 65 years or older

[ ] most patients aged 65 years or older

[ ] some patients aged 65 years or older

[ ] few patients aged 65 years or older

[ ] no patients aged 65 years or older

1. Now specifically about ECGs, when an ECG is required for a patient, how do you usually obtain it? [Please select all that apply]

[ ] I use a 12-lead ECG machine in my/ our practice

[ ] I refer to a pathology service

[ ] I refer to a cardiology service

[ ] I use a service at a local hospital

[ ] I use a mobile handheld ECG device

[ ] Other – please specify______________

1. When an ECG is required for a patient, how time-consuming is obtaining a conventional 12-lead ECG?

[ ] Extremely time-consuming

[ ] Very time-consuming

[ ] Moderately time-consuming

[ ] Slightly time-consuming

[ ] Not time-consuming at all

1. Do you consider assessing your patients for arrhythmias, other than atrial fibrillation?

Yes/ No

If yes, which of the following arrhythmias do you look for? (Please select all that apply)

[ ] Complete heart block

[ ] 2^nd^ degree heart block

[ ] Left bundle branch block

[ ] Trifascicular block

[ ] Long QT

[ ] Other, please specify

1. How confident are you in diagnosing the following arrhythmias or ECG abnormalities on 12-lead ECG?
2. Atrial fibrillation

| Extremely confident | Very confident | Moderately confident | Mildly confident | Not confident |
| --- | --- | --- | --- | --- |

1. Complete (3^rd^ degree) Heart Block

| Extremely confident | Very confident | Mderately confident | Mildly confident | Not confident |
| --- | --- | --- | --- | --- |

1. Distinguish between Mobitz I (Wenckebach) and Mobitz II second degree heart block

| Extremely confident | Very confident | Moderately confident | Mildly confident | Not confident |
| --- | --- | --- | --- | --- |

1. Left bundle branch block

| Extremely confident | Very confident | Moderately confident | Mildly confident | Not confident |
| --- | --- | --- | --- | --- |

1. Trifascicular block

| Extremely confident | Very confident | Moderately confident | Mildly confident | Not confident |
| --- | --- | --- | --- | --- |

1. Prolonged QT-interval

| Extremely confident | Very confident | Moderately confident | Mildly confident | Not confident |
| --- | --- | --- | --- | --- |

1. Are you aware of the automatic ECG interpretation provided by a conventional 12-lead ECG machine? Yes/ No

If yes, how helpful is the automatic ECG interpretation provided by a conventional 12-lead machine?

[ ] Extremely helpful

[ ] Very helpful

[ ] Moderately helpful

[ ] Slightly helpful

[ ] Not helpful at all

1. Have you attended training on ECG interpretation in the last 3 years? Yes/ No

If yes, please specify the type of training (such as one-hour tutorial, one-day seminar, two-day workshop, online course): _________________

1. Do you use a mobile device to obtain a single-lead ECG on patients?

[ ] Regularly

[ ] Sometimes (such as six-monthly)

[ ] Occasionally

[ ] Never but I am contemplating using one

[ ] Never and I am not contemplating using one

1. Whether or not you use a mobile health device, how confident are you that a mobile handheld ECG device can provide adequate quality information to detect:
2. Atrial fibrillation

| Extremely confident | Very confident | Moderately confident | Mildly confident | Not confident |
| --- | --- | --- | --- | --- |

1. Complete heart block

| Extremely confident | Very confident | Moderately confident | Mildly confident | Not confident |
| --- | --- | --- | --- | --- |

1. Prolonged QT

| Extremely confident | Very confident | Moderately confident | Mildly confident | Not confident |
| --- | --- | --- | --- | --- |

About you:

1. Which best describes you? (Please select one)

[ ] GP with FRACGP

[ ] GP with FACRRM

[ ] GP with FRACGP and FACRRM

[ ] GP Registrar

[ ] Other (please specify)____

1. How long is it since you commenced working in general practice?

[ ] less than 1 year

[ ] 1- 4 years

[ ] 5- 9 years

[ ] 10- 19 years

[ ] 20 years and more

1. Your gender:

[ ] male

[ ] female

[ ] rather not say

1. Approximately how many hours per week do you usually work in general practice?

[ ] 1-8 hours

[ ] 9-16 hours

[ ] 17-24 hours

[ ] 25-32 hours

[ ] 33-40 hours

[ ] > 40 hours

1. What is the postcode of your main practice? ___________
2. If ECG interpretation training were available, how would you prefer to receive the training? [Please select one]

[ ] hardcopy such as “RACGP Check magazine”

[ ] webinar (seminar via the internet)

[ ] online education module

[ ] face to face workshop

[ ] other, specify __________________

1. How did you **first** hear about this survey? [Please select one]

[ ] RACGP website

[ ] RACGP newsletter

[ ] ACRRM website

[ ] ACRRM newsletter

[ ] GP Registrar Training Organization

[ ] Primary Health Network

[ ] GP supervisor

[ ] GP peer

[ ] GP Conference

[ ] University

[ ] Researcher

[ ] Other, please specify ____________

**THIS IS THE END OF THE SURVEY. THANK YOU.**

- Can we contact you to consider participating in a one-on-one interview about mobile health devices or to test a mobile ECG device? ---**Yes/ No**
- Would you like to attend ECG interpretation training if we plan to deliver such training? **Yes/ No**
- Do you want to participate in the lucky draw? **Yes/ No**

[Participants who answer “yes” to any of the above three questions will be taken to a separate survey page to provide their email address so that their email-address will not be linked to their answers to the survey.]

If you would like to participate in the lucky draw or attend an interview or receive notification regarding ECG interpretation training, please provide your email address:

**__________________________@_________________**

Please note that even if you consent to be contacted you can still decline to participate at any time.

If you would like to give us suggestion or ask a question, you may email [kam.wong@sydney.edu.au](mailto:kam.wong@sydney.edu.au)
